# Effects of ambient particulate matter on a reconstructed human corneal epithelium model

**DOI:** 10.1038/s41598-021-82971-1

**Published:** 2021-02-09

**Authors:** Ryota Ko, Masahiko Hayashi, Miho Tanaka, Tomoaki Okuda, Chiharu Nishita-Hara, Hiroaki Ozaki, Eiichi Uchio

**Affiliations:** 1 Department of Ophthalmology, Faculty of Medicine, Fukuoka University, 7-45-1 Nanakuma, Jonan-ku, Fukuoka, 814-0180 Japan; 2 Department of Earth System Science, Faculty of Science, Fukuoka University, 8-19-1 Nanakuma, Jonan-ku, Fukuoka, 814-0180 Japan; 3 Kobayashi Pharmaceutical Co., Ltd., 1-30-3, Toyokawa, Ibaraki, Osaka 567-0057 Japan; 4 Department of Applied Chemistry, Faculty of Science and Technology, Keio University, 3-14-1 Hiyoshi, Kohoku, Yokohama 223-8522 Japan; 5 Fukuoka Institute for Atmospheric Environment and Health, Fukuoka University, 8-19-1 Nanakuma, Jonan-ku, Fukuoka, 814-0180 Japan

**Keywords:** Cell death and immune response, Corneal diseases

## Abstract

We evaluated the effects of ambient particulate matter (PM) on the corneal epithelium using a reconstructed human corneal epithelium (HCE) model. We collected two PM size fractions [aerodynamic diameter smaller than 2.4 µm: PM_0.3–2.4_ and larger than 2.4 µm: PM_>2.4_] and exposed these tissues to PM concentrations of 1, 10, and 100 µg/mL for 24 h. After exposure, cell viability and interleukin (IL) IL-6 and IL-8 levels were determined, and haematoxylin and eosin and immunofluorescence staining of the zonula occludens-1 (ZO-1) were performed on tissue sections. In addition, the effects of a certified reference material of urban aerosols (UA; 100 µg/mL) were also examined as a reference. The viability of cells exposed to 100 μg/mL UA and PM_>2.4_ decreased to 76.2% ± 7.4 and 75.4% ± 16.1, respectively, whereas PM_0.3–2.4_ exposure had a limited effect on cell viability. These particles did not increase IL-6 and IL-8 levels significantly even though cell viability was decreased in 100 μg/mL UA and PM_>2.4_. ZO-1 expression was reduced in a dose-dependent manner in all groups. Reconstructed HCE could be used as an in vitro model to study the effects of environmental PM exposure on ocular surface cell viability and inflammation.

## Introduction

Air pollution represents one of the greatest environmental risks to health. Especially, the adverse health effects of ambient particulate matter (PM) have recently been reported worldwide^1–5^. Ambient PM is composed of various kinds of particles with different size, chemical composition, and morphology. Particle size is a very important property of PM because its deposition rate in vivo changes depending on its size. Only very small particles can be inhaled and deposited in the lungs; therefore, the US national health standards for the quality of ambient air are based on the mass concentration of “inhalable particles”, defined to include particles with an aerodynamic diameter of smaller than 10 µm. The emission source and chemical composition of ambient PM also vary according to the size. Particles between approximately 2.5 and 10 μm in diameter, known as coarse particles, typically contain resuspended dust from roads, industrial activities, and soils and biological materials such as pollen grains and bacterial fragments, while particles smaller than approximately 2.5 μm in diameter, known as fine particles or PM_2.5_, are typically composed of nitrate, sulphate, ammonium, elemental carbon (EC), a large number of organic compounds and trace metals^6^. Previous reports revealed that PM has adverse biological effects such as decreased cell viability or the secretion of pro-inflammatory cytokines^7,8^. However, the chemical components and physical properties that determine ambient PM toxicity are not sufficiently understood thus far.

Epidemiologically, ambient PM affects ocular surface health, leading to conditions such as allergic conjunctivitis^9,10^, in addition to increasing the risk of death from cardiovascular and respiratory illnesses^11^. Several studies have addressed the effects of house dust or diesel exhaust particles (DEP), one of the major forms of PM in urban air pollution, on the ocular mucosa^12–14^. However, few studies have investigated the effects of ambient PM on ocular surfaces. One of the reasons for this lack of evidence is that a large amount of PM is required to evaluate the adverse effects on health through in vitro studies. Unfortunately, it is difficult to collect a sufficient amount of PM by extraction using the conventional filter collection method.

Previous studies have shown the relationship of the ocular surface with DEP using human corneal or conjunctival conventional two-dimensional cell cultures^12,13^. DEP influence cell viability, and moreover tight junctions (TJ) such as the zonula occludens-1 (ZO-1); however, conventional two-dimensional cell cultures may not be able to predict biological responses as essential cellular functions in living tissues are missing^15^. Cao et al. demonstrated that reconstructed human corneal epithelial (HCE) tissue cultures can be used to study the eye-irritating and inflammatory potential of lipopolysaccharides and house dust^14^. This kind of model closely resembles native corneal epithelium^16^. The applicability of this model in eye irritation testing using chemicals with established eye-irritating potential has satisfactory validated specificity and reproducibility^17^. Therefore, a recently developed reconstructed HCE tissue culture model may be a useful tool for in vitro toxicological studies. Our search of previously published papers found no article that reported the toxicity of ambient PM in reconstructed HCE.

The aim of this study was to evaluate the effect of ambient PM exposure on the corneal epithelium in vitro using a reconstructed HCE model. In this study, using a cyclone technique recently developed by Okuda et al., sufficient amounts of two size fractions of ambient PM (aerodynamic diameter smaller than 2.4 µm: PM_0.3–2.4_ and larger than 2.4 µm: PM_>2.4_) were collected from the urban atmosphere in Japan^18^. In addition, we used an environmental certified reference material of urban aerosols developed by the National Institute of Environmental Studies, Japan (NIES CRM No.28 Urban Aerosols: UA) as a reference for comparison with previous studies. We analysed cell viability, secretion of proinflammatory cytokines, and histological changes in the TJ of HCE, and clarified that PM was harmful to the ocular surface in terms of these points.

## Results

### Characterisation of PM and urban aerosols (UA)

**Figure 1:**
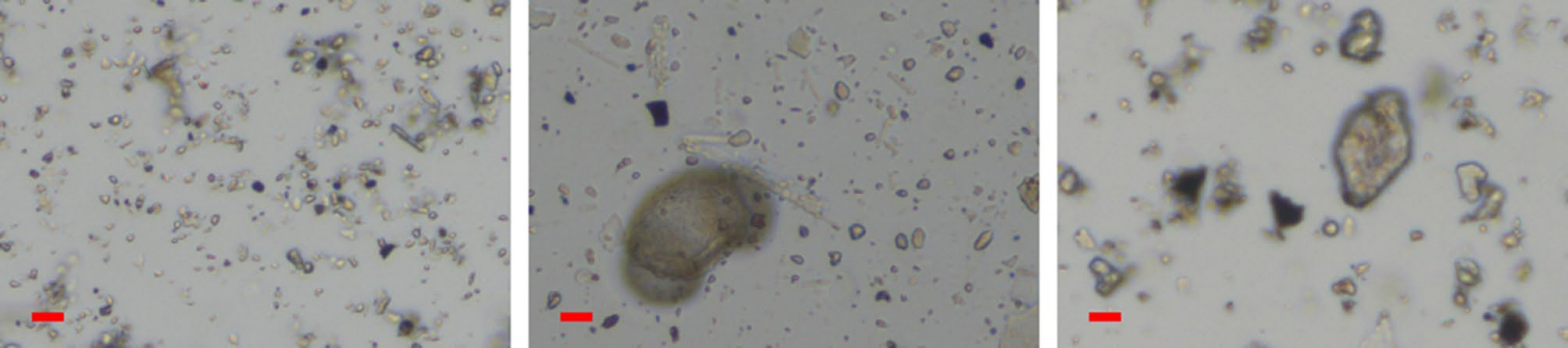
Morphology of PM_0.3–2.4_ (left), PM_>2.4_ (centre), and UA (right). The length of the bar in each photograph is 10 µm. *PM*_*0.3–2.4*_ particle matter 0.3–2.4 µm, *PM*_>*2.4*_ particle matter larger than 2.4 µm, *UA* urban aerosols.

Figure 1 shows the morphology of PM_0.3–2.4_, PM_>2.4_ and UA. Particles with diameter > 5 µm were rarely found in PM_0.3–2.4_, whereas many particles with diameter > 5 µm were found in PM_>2.4_. Even large particles with size > 20 µm, which were considered to be pollen, were also found in PM_>2.4_. On average, UA contained larger particles than PM_0.3–2.4_ and PM_>2.4_. The geometric particle sizes of PM_0.3–2.4_, PM_>2.4_, and UA observed in this study are consistent with those reported in previous studies^19,20^.

The concentration of endotoxin in PM_0.3–2.4_, PM_>2.4_ and UA was 0.140, 0.060, and 0.037 EU/mg, respectively (Table 1). The concentration of β-glucan was 5163, 7949, and 42,405 pg/mg, respectively. The oxidative potential measured by dithiothreitol (DTT) assay of PM_0.3–2.4_, PM_>2.4_ and UA was 21.6, 18.3 and 30.8 pmol/min/µg, respectively (Table 1). The concentrations of water-soluble ions and elements in PM_0.3–2.4_, PM_>2.4_, and UA are shown in Table 1. Briefly, concentrations of SO_4_^2−^, K^+^, Mg^2+^, and Ca^2+^ were higher in UA than in PM_0.3–2.4_ and PM_>2.4_. As for elements, PM_>2.4_ tended to have higher concentrations of Si, Cl, K, Fe, and Cu than the others, whereas the concentrations of S, Ca, Zn, and Pb were markedly higher in UA than in the others. As for carbonaceous components, PM_0.3–2.4_ and PM_>2.4_ contained more organic carbon (OC) than EC, whereas UA contained more EC than OC. The concentration of EC in UA was overwhelmingly higher than that in PM_0.3–2.4_ and PM_>2.4_.

**Table 1:** Concentrations of endotoxin, β-glucan, ions, elements, and carbonaceous components in particle matters and oxidative potential.

		PM_0.3–2.4_	PM_>2.4_	UA	Unit
Endotoxin		0.140	0.060	0.037	EU/mg
β-glucan		5163	7949	42,405	pg/mg
Ions	Cl^−^	5152	11,691	5550	ppm
	NO_3_^−^	61,570	74,021	18,010	
	SO_4_^2−^	36,565	18,032	79,080	
	Na^+^	13,918	16,140	2600	
	NH_4_^+^	3494	1502	2920	
	K^+^	1768	735	2870	
	Mg^2+^	195	542	2910	
	Ca^2+^	5295	7753	41,090	
Elements	Mg	11,280	13,077	14,000*	ppm
	Al	44,106	60,357	50,400*	
	Si	120,368	187,994	149,000*	
	P	1090	1386	1450*	
	S	11,821	8744	39,100*	
	Cl	12,022	58,954	8070*	
	K	15,042	24,116	13,700*	
	Ca	20,742	42,554	66,900*	
	Ti	1990	3380	2920*	
	V	82	95	73*	
	Cr	115	90	66*	
	Mn	585	931	686*	
	Fe	23,181	36,381	29,200*	
	Ni	66	58	64*	
	Cu	91	141	104*	
	Zn	538	524	1140*	
	Pb	122	115	403*	
Carbonaceous components	EC	9255	13,573	68,000**	ppm
	OC	99,206	70,508	55,000**	
Oxidative potential***		21.6	18.3	30.8	pmol/min/µg

*Refer to Mori et al.^20^.

**Refer to Okuda^53^.

***Measured by dithiothreitol assay.

### Biological effects of PM on reconstructed HCE models

**Figure 2:**
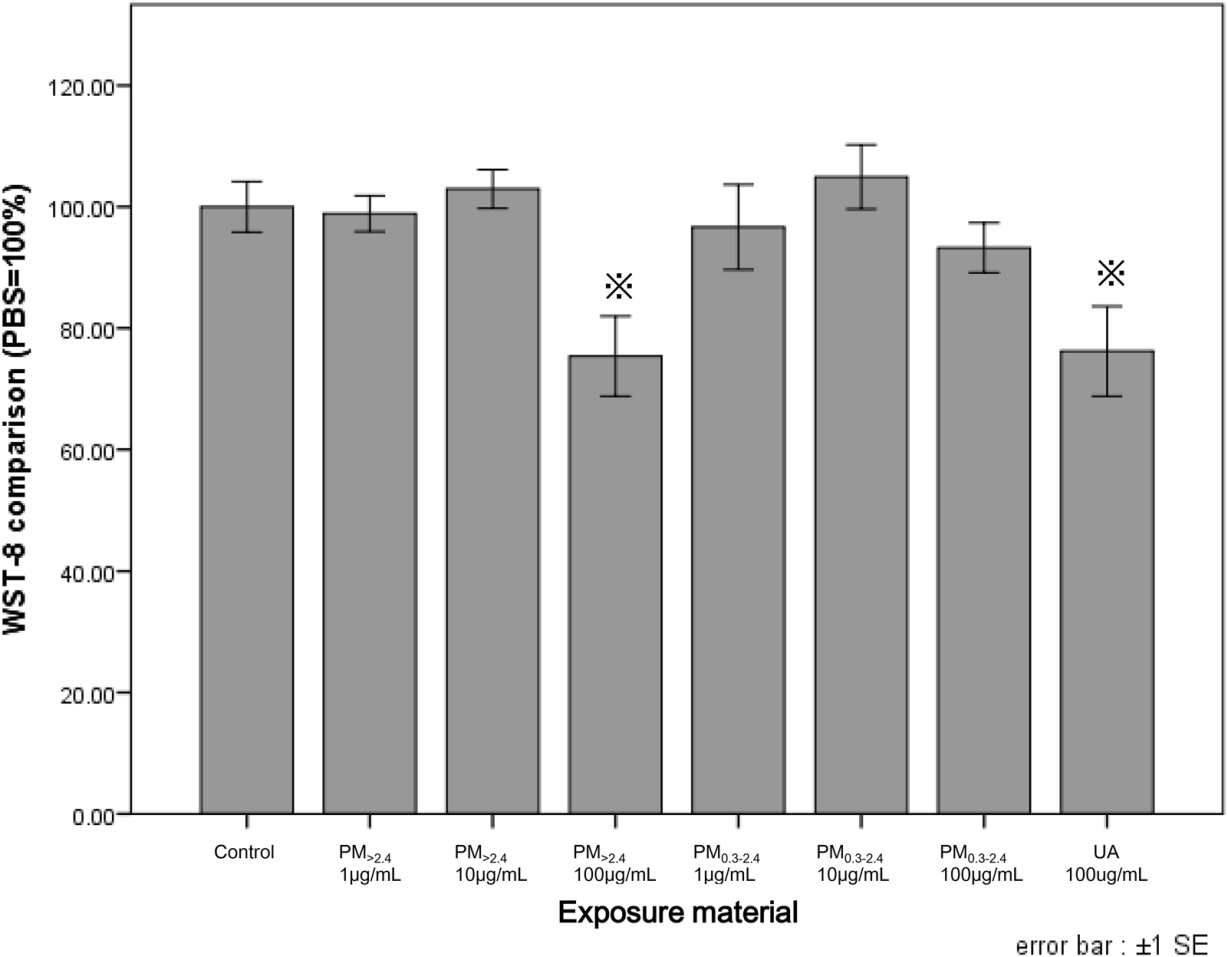
Effects of ambient particles and urban aerosols on reconstructed HCE tissues. Data are expressed as the percentage of cell viability based on the control (0 µg/mL). Each data are mean ± SE of 6 PM_>2.4_, 3 PM_0.3–2.4_, 9 UA and 9 control individual cultures. This figure was created using the software SPSS Statistics 24.0 (https://www.ibm.com/products/spss-statistics?lnk=STW_US_STESCH_P1_BLK&lnk2=trial_SPSSstat&lot=1&pexp=def&psrc=none&mhsrc=ibmsearch_a&mhq=spss). ^*^p < 0.05 vs. control (0 µg/mL). *HCE* human corneal epithelial, *SE* standard error of the mean, *PM*_>*2.4*_ particle matter larger than 2.4 µm, *PM*_*0.3–2.4*_ particle matter 0.3–2.4 µm, *UA* urban aerosols.

**Figure 3:**
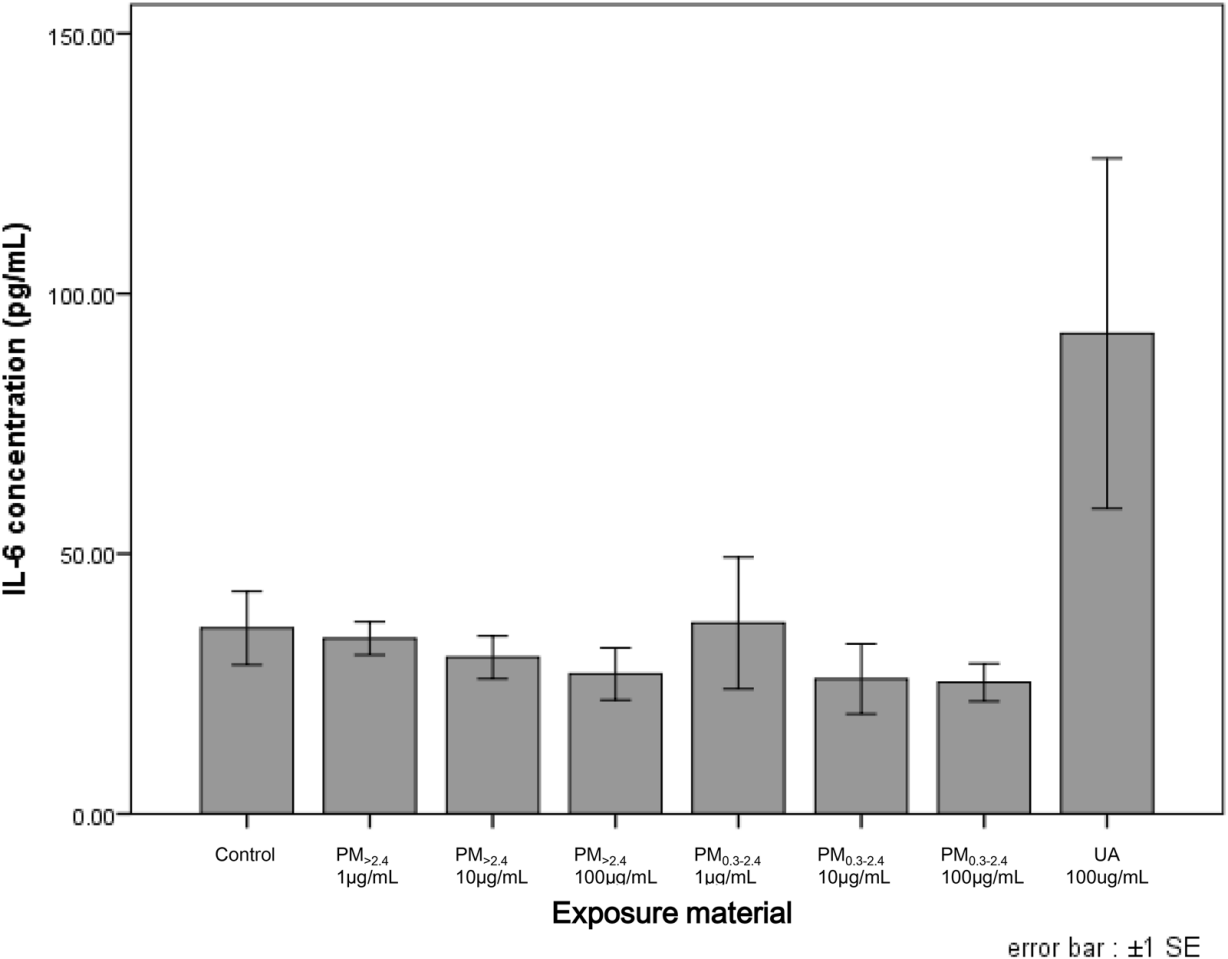
Production of IL-6 from reconstructed human corneal epithelial tissues following exposure to ambient particles and urban aerosols. Each data are mean ± SE of 6 PM_>2.4_, 3 PM_0.3–2.4_, 8 UA and 8 control individual cultures. This figure was created using the software SPSS Statistics 24.0 (https://www.ibm.com/products/spss-statistics?lnk=STW_US_STESCH_P1_BLK&lnk2=trial_SPSSstat&lot=1&pexp=def&psrc=none&mhsrc=ibmsearch_a&mhq=spss). *IL* interleukin, *SE* standard error of the mean, *PM*_>*2.4*_ particle matter larger than 2.4 µm, *PM*_*0.3–2.4*_ particle matter 0.3–2.4 µm, *UA* urban aerosols.

**Figure 4:**
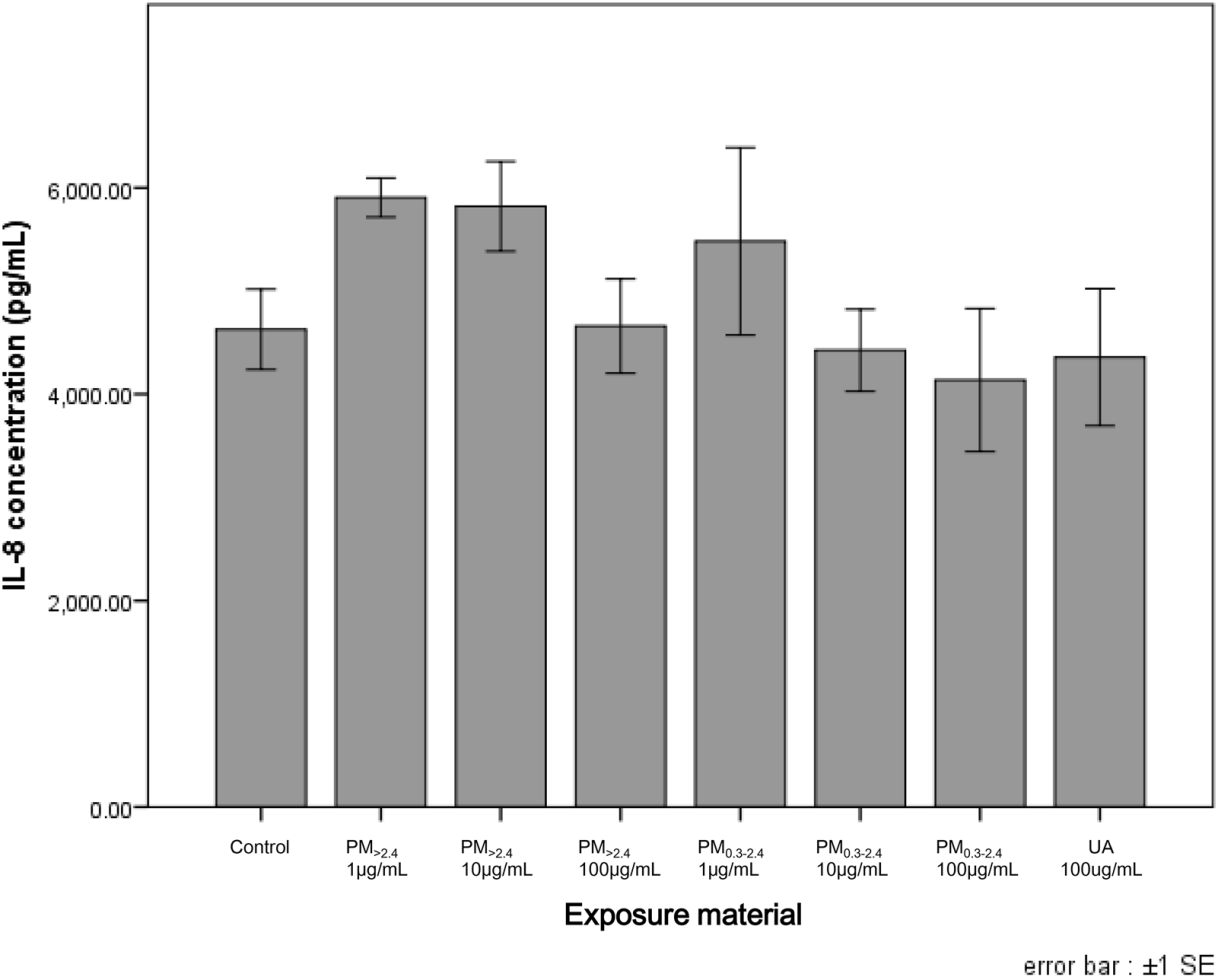
Production of IL-8 from reconstructed human corneal epithelial tissues following exposure to ambient particles and urban aerosols. Data are mean ± SE of 6 PM_>2.4_, 3 PM_0.3–2.4_, 6 UA and 6 control individual cultures. This figure was created using the software SPSS Statistics 24.0 (https://www.ibm.com/products/spss-statistics?lnk=STW_US_STESCH_P1_BLK&lnk2=trial_SPSSstat&lot=1&pexp=def&psrc=none&mhsrc=ibmsearch_a&mhq=spss). *IL* interleukin, *SE* standard error of the mean, *PM*_>*2.4*_ particle matter larger than 2.4 µm, *PM*_*0.3–2.4*_ particle matter 0.3–2.4 µm, *UA* urban aerosols.

The effects of each PM on the reconstructed HCE model are shown in Figs. 2, 3 and 4. Conversion of WST-8 was significantly decreased in PM_>2.4_ 100 µg/mL and UA 100 µg/mL as compared with that observed in the control group (*p* < 0.05) (Fig. 2). Meanwhile, PM_0.3–2.4_ 1 µg/mL and 100 µg/mL demonstrated a very limited reduction compared with the control.

Figures 3 and 4 show the concentrations of cytokines/chemokines in the medium after exposure to PM. A difference in the concentration of IL-6 between these particles and the control was observed only at UA 100 µg/mL; however, this difference was not statistically significant. For IL-8, the concentration was similar between UA 100 µg/mL and the control. PM_>2.4_ 1 µg/mL and PM_0.3–2.4_ 1 µg/mL exhibited the highest concentration of IL-8, followed by a decrease in a dose-dependent manner in each group. The concentration of IL-8 in PM_>2.4_ 100 µg/mL and PM_0.3–2.4_ 100 µg/mL was not different from that in the control.

### Histological changes in reconstructed HCE model after exposure

**Figure 5:**
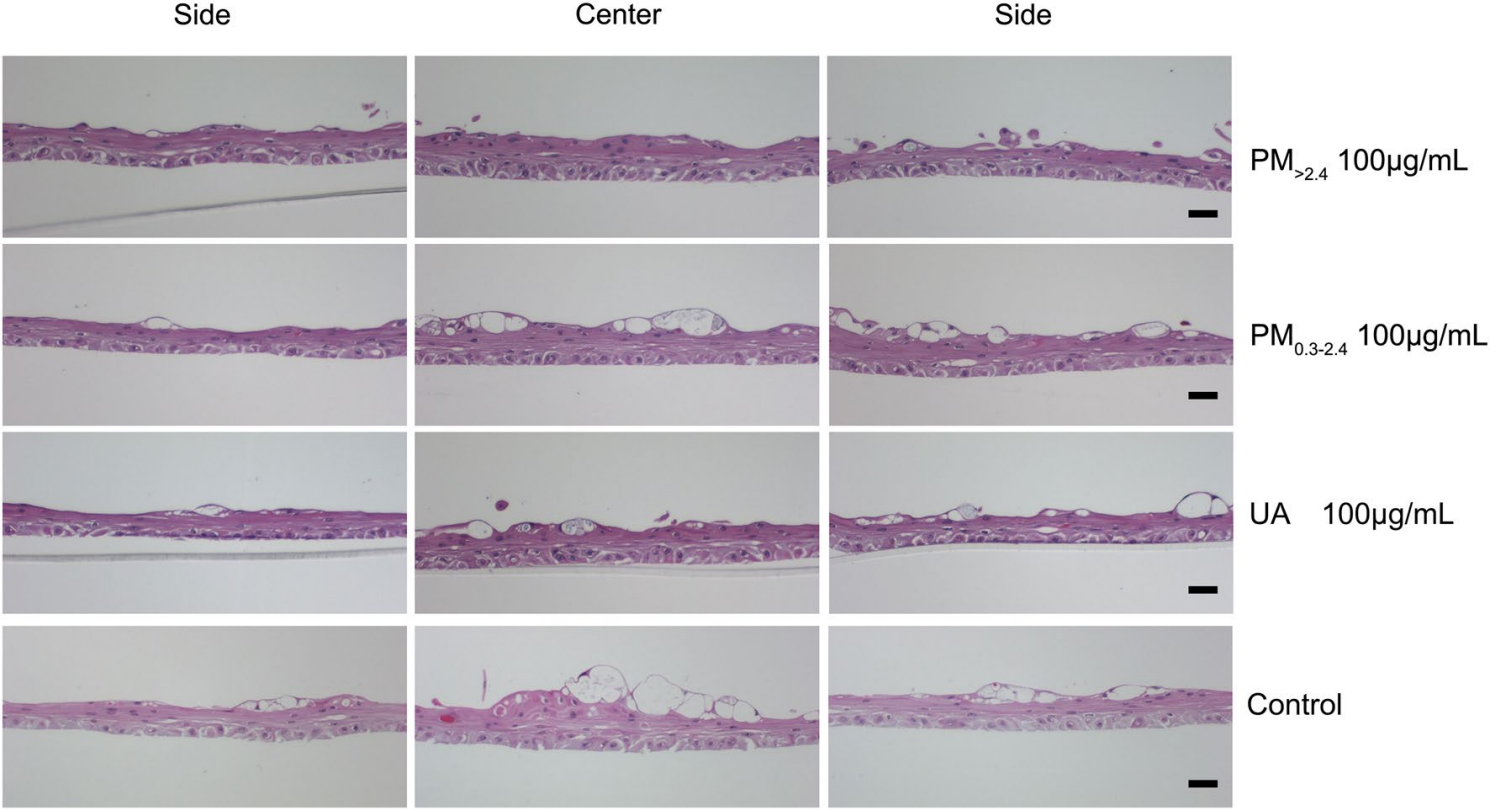
Representative histological images (hematoxylin and eosin staining, ×100 magnification, both sides and center of each model). Reconstructed human corneal epithelial tissues after 24-h exposure to PM_>2.4_ 100 µg/mL, PM_0.3–2.4_ 100 µg/mL, UA 100 µg/mL, and control. The length of the bar in each photograph is 50 µm. *PM*_>*2.4*_ particle matter larger than 2.4 µm, *PM*_*0.3–2.4*_ particle matter 0.3–2.4 µm, *UA* urban aerosols.

Morphological changes in the HCE model after exposure to control, PM_>2.4_ 100 µg/mL, PM_0.3–2.4_ 100 µg/mL and UA 100 µg/mL were examined by histological studies (Fig. 5). The captured photographs were divided into three parts (both sides and the centre of the model). Both sides of each model tended to be thinner than the centre, and there was no significant difference in the whole tissue area among these models (data not shown).

**Figure 6:**
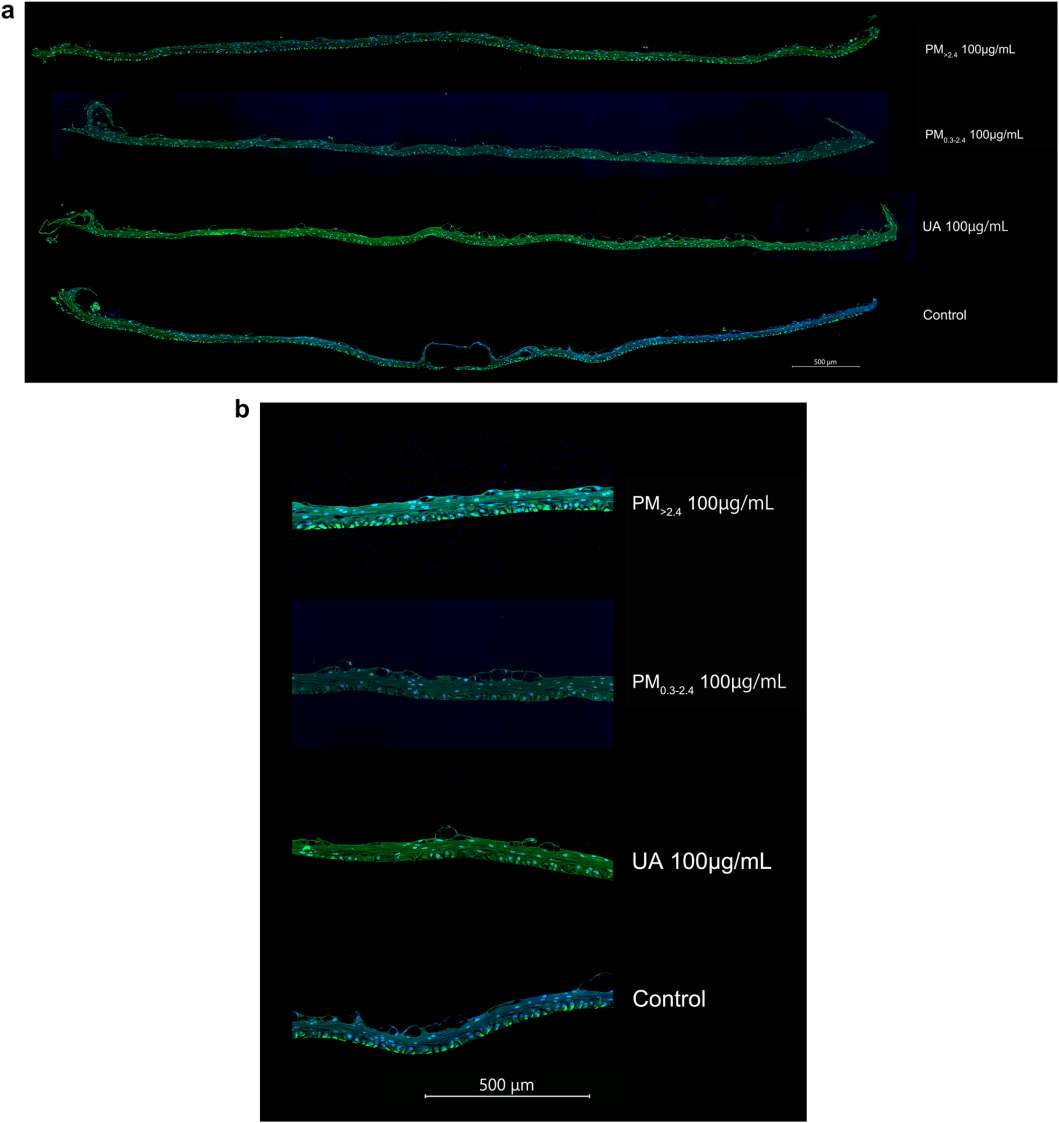
Immunohistological findings of expression of zonula occludens-1 (ZO-1) (**a** whole image, **b** enlarged image). ZO-1 was analysed through immunostaining of reconstructed human corneal epithelial tissues treated with ambient particles and urban aerosols. Strong fluorescence intensity indicates expression of ZO-1. These are shown at the bottom of the tissues mainly among all groups. The control had the strongest intensity, whereas PM_0.3–2.4_ 100 µg/mL had the weakest intensity. *ZO-1* zonula occludens-1, *PM*_>*2.4*_ particle matter larger than 2.4 µm, *PM*_*0.3–2.4*_ particle matter 0.3–2.4 µm, *UA* urban aerosols.

**Figure 7:**
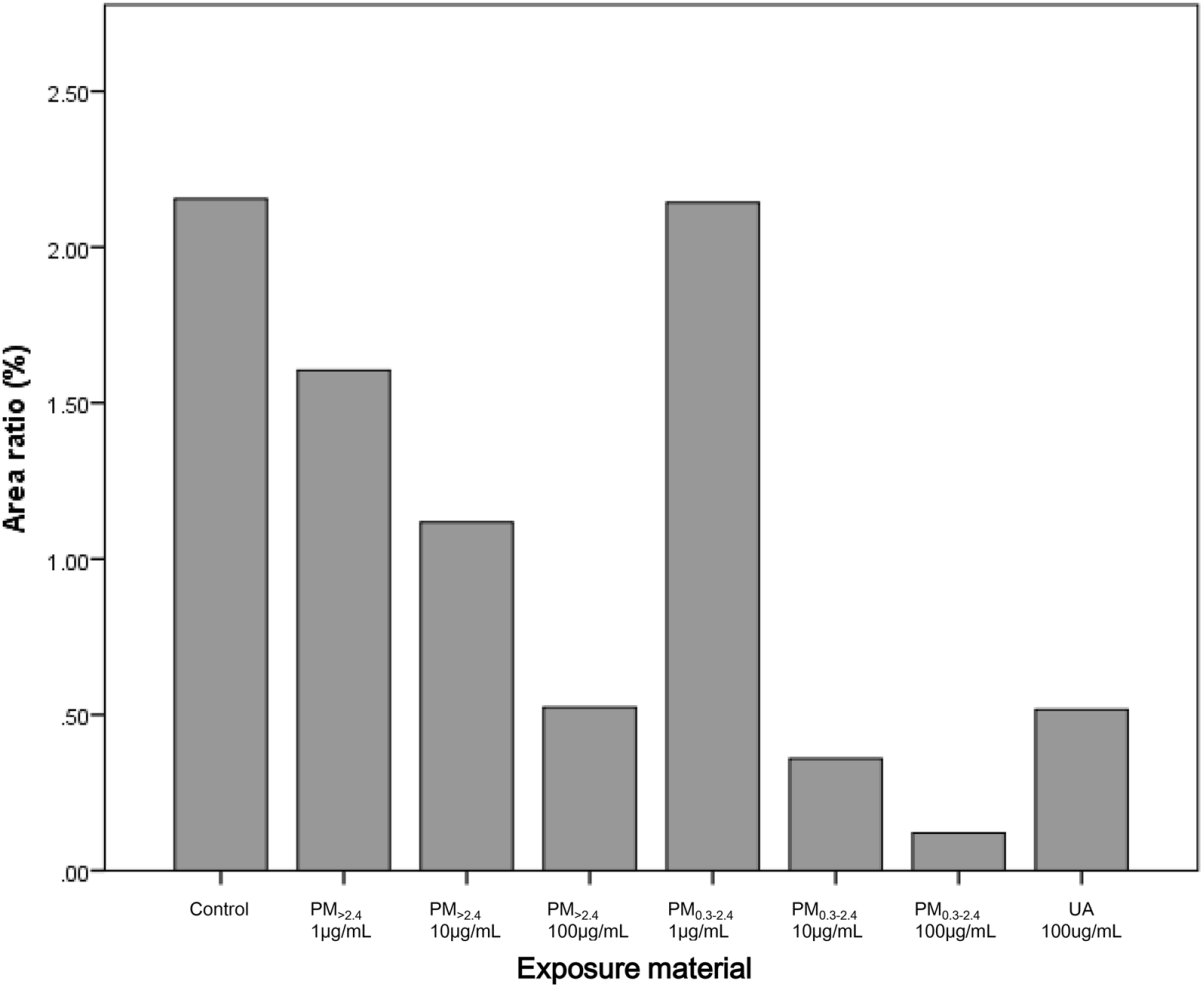
Area ratio of expression of ZO-1 in representative immunostained reconstructed human corneal epithelial tissues. This figure was created using the software SPSS Statistics 24.0 (https://www.ibm.com/products/spss-statistics?lnk=STW_US_STESCH_P1_BLK&lnk2=trial_SPSSstat&lot=1&pexp=def&psrc=none&mhsrc=ibmsearch_a&mhq=spss). *ZO-1* zonula occludens-1, *PM*_>*2.4*_ particle matter larger than 2.4 µm, *PM*_*0.3–2.4*_ particle matter 0.3–2.4 µm, *UA* urban aerosols.

Immunofluorescence analysis revealed decreased ZO-1 expression in the reconstituted HCE model that was exposed to PM_>2.4_, PM_0.3–2.4_, and UA in a dose-dependent manner (Figs. 6, 7). PM_>2.4_ exerted a greater adverse effect on this tissue than PM_0.3–2.4_ at a concentration of 1 µg/mL, whereas PM_0.3–2.4_ 100 µg/mL resulted in the greatest decrease in ZO-1 expression. Exposure to PM_>2.4_ 100 µg/mL and UA 100 µg/mL showed a similar effect on HCE tissue.

## Discussion

The findings of this study revealed that only a high-dose of PM_>2.4_ and UA significantly decreased cell viability in a reconstructed HCE model, and these particles did not induce the production of proinflammatory cytokines in this tissue. These findings indicate that the most harmful particles to reconstructed HCE tissue may be UA and PM_>2.4_, followed by PM_0.3–2.4_, among the particulate matters used in our experiments. On the other hand, all types of particles, especially PM_0.3–2.4_, decreased the expression of ZO-1 in a dose-dependent manner.

Previous studies have investigated the toxicity of PM in vitro^12–14,19^. It has been reported that exposure of the ocular surface to DEP increased the expression of inflammatory factors in the human conjunctiva^12^. Human corneal and conjunctival epithelial cells incubated with DEP showed a decrease of cell viability, increase of IL-6 secretion and decrease of IL-8 secretion^13^. Cao et al. reported decreased cell viability in reconstructed HCE incubated with high-dose house dust (15 and 30 mg/mL) and increased levels of IL-8 and IL-1β^14^. On the other hand, Onishi et al. reported that ambient fine and coarse particles, collected using the same cyclone technique as used in our study, decreased the viability of nasal epithelial cells. However, detectable cytokine release in nasal epithelial cells was not induced by any particle. In addition, bronchial epithelial cells did not show a decrease in viability following exposure to both particles; however, these particles increased the release of IL-6 in a dose-dependent manner^19^. Thus, it is presumed that the effects of PM depend on the type of cell or exposed PM characteristics. In our experiment, there were no differences in the expression of IL-6 and IL-8 even though viability decreased significantly at PM_>2.4_ 100 µg/mL and UA 100 µg/mL. Onishi et al. also considered that the reason these particles decreased only the viability of nasal epithelial cells but did not induce pro-inflammatory cytokines was that these cells have less Toll-like receptor (TLR) expression and greater distribution of Toll-interacting protein. These features can maintain nasal homeostasis even though these epithelial cells in the nasal cavity are always exposed to the external environment and various bacteria or chemicals therein. The same is true of the ocular surface. The ocular surface, including corneal epithelial cells, is always exposed too; however, the eye is extremely delicate and intolerant of the distortion caused by inflammation. Thus, the eye needs immune privilege to maintain function. Medawar considered that the absence of lymphatic-drainage pathways in the eye is important for shielding ocular antigens from the immune system^21^. Moreover, no corneal cells express MHC class II antigens, and expression of MHC class I antigens is reduced^22^. According to previous reports, incubation of human conventional two-dimensional corneal epithelial cells with DEP and reconstructed HCE models with high-dose house dust influenced cell viability and changed the levels of some pro-inflammatory cytokines^13,14^. However, these conventional corneal model cells were monolayer, rather than reconstructed; therefore, they would be more easily affected by exposed materials. Moreover, regarding the experiments using reconstructed HCE models with high-dose house dust, the concentrations of exposed materials were so much higher compared to our experiment that these house-dust particles influenced cell viability and the secretion of pro-inflammatory cytokines. These may be the reasons that only viability decreased but pro-inflammatory cytokines were not induced in our experiment.

When we consider what in PM might be related to its toxicity in this reconstructed HCE, several studies that have examined the associations between ambient PM and ocular toxicity epidemiologically might be useful. Chang et al. reported that coarse particles are more strongly associated with nonspecific conjunctivitis than are fine particles. In their study, only coarse particles had a significant impact on the number of outpatient visits for nonspecific conjunctivitis^10^. Experimental and epidemiological studies have demonstrated that coarse particles may exhibit a similar or higher proinflammatory potential than fine particles and lead to adverse pulmonary responses^23–25^. These findings might be attributable to the stronger influence of microbial factors (e.g., endotoxins, β-glucan, and pollen) in coarse particles than those in fine particles. Previous studies have shown that endotoxin and β-glucan are associated with the inflammatory effects of ambient PM in vitro as well as in vivo^26–28^. In the present study, pollen was found in PM_>2.4_ (Fig. 1). Pollens may be one of the factors that can explain why PM_>2.4_ had a greater adverse effect on this model, although other factors were not considered completely.

On the other hand, UA caused a decrease of cell viability too. One of the reasons might be the high concentration of glucan in UA. The glucan test is a US Food and Drug Administration-approved quantitative assay that is used to aid in the detection of invasive fungal infections^29^. A glucan value of < 60 pg/mL is considered a negative result^30^. Accordingly, it is suggested that exposure to UA leads to a decrease in cell viability, which is induced by the glucan contained in UA.

We showed that ZO-1 expression declined in a dose-dependent manner following exposure to these particles, especially PM_0.3–2.4_. TJs comprise a complex plaque of proteins, such as claudins and occludins, which are attached by the ZO proteins to the cytoskeleton of actin protein across the cell membrane^31^. It has been reported that DEP modulates the permeability of vascular endothelial cells or alveolar epithelial cells by downregulating the expression of ZO-1^32,33^. Furthermore, previous studies revealed that DEP and fine particles reduced the expression of ZO-1 in human nasal epithelial cells^34,35^. It is reported that nasal epithelial barrier disruption is caused by oxidative stress. In this study, the PM with the highest oxidative potential measured by DTT assay was UA, followed by PM_0.3–2.4_ and PM_>10_; however, PM_0.3–2.4_ had the greatest influence on TJs in reconstructed HCE tissue. According to this result, there may be other reasons why these particles reduce the expression of ZO-1. Interestingly, exposure to PM_0.3–2.4_ decreased ZO-1 expression but did not influence cell viability and the secretion of pro-inflammatory cytokines. In previous reports, the same situation was observed in nasal epithelial cells with DEP exposure^19^. Disruption of epithelial barrier function is associated with severe corneal damage in severe allergic eye diseases^36^. Our results suggest that these particles allow the invasion of certain allergens into corneal epithelial cells, exacerbating allergic reactions on the ocular surface even though PM_0.3–2.4_ did not affect cell viability or the secretion of pro-inflammatory cytokines.

Previous studies have reported that the chemical components of PM, such as ions, elements, and carbons, influence cell viability, the production of cytokines, and the expression of ZO-1. Elements such as Al, Ca, Si, Fe, Zn, Cr, Mn, V and Cu induce toxicity in airway epithelial cells^37–39^. Another report showed that engineered nanoparticles (ENPs) such as copper oxide (CuO) and zinc oxide (ZnO) decreased the cell viability of human corneal limbal epithelial cells, because these ENPs can partially dissolve in culture media and release ions, and these ions may inhibit cell viability^40^. On the other hand, Xiang et al. indicated that Zn, Cu, Mn, Pb, and Cr, which are included in house dust and office dust, reduced ZO-1 immunoreactivity^41^, whereas a previous study concisely concluded that sulphate and nitrate aerosols have little biological potency in normal humans and animals^42^. Moreover, in spite of the fact that OC and EC constitute a considerable part of particle mass, there is great uncertainty about their precise contributions to health effects, owing primarily to its complex, heterogenous nature, which is not well characterized in most geographical settings^43^.

Finally, as we mentioned above, there is some interest in oxidative potential which is considered a health-related factor of PM, especially in fine particles^44–46^. Although much in known about the pathway activated by reactive oxygen species (ROS), there is no direct evidence on the mechanisms leading to an increase in ROS levels in cells exposed to PM^45^. However, elements, organic compounds, and bacterial endotoxin are considered to have a role in the generation of oxidative potential^47–50^. In our study, the PM with the most oxidative potential was UA, followed by PM_0.3–2.4_, and PM_>2.4_. It is difficult to clarify the main factor in oxidant potential; however, it may be determined by various factors interacting with each other.

There are some limitations of this study. Firstly, the ocular surface possesses specific defense components, such as epithelial glycocalyx. Glycocalyx plays a significant role in allergic reactions on the ocular surface^51^. Tau et al. reported that a decrease of mucin expression observed in corneal cells might result in enlargement of areas exposed to contact with DEP in the cornea^13^. In this study, we did not evaluate the function of glycocalyx histologically. Second, we could not analyse the correlation coefficient of the PM constituents and cell viability and the expression of ZO-1 because the sample number of PM was too small. Further studies are needed with greater variety of these particles collected with the cyclone technique.

In summary, the results of our in vitro study have revealed the eye-irritating potential of fine and coarse particles in the atmosphere collected through the cyclone technique, suggesting decreased HCE viability and ZO-1 expression. These effects vary between fine and coarse particles, which could be attributable to differences in the concentrations of biological materials, chemical composition, and particle size. Reconstructed HCE tissue may reflect the true reaction of the cornea more than do conventional cornea epithelial models, and it could be used as an in vitro model for the assessment of ocular surface change induced through exposure to environmental PM. Further studies are warranted to determine the mechanism through which the properties of PM affect the ocular surface.

## Methods

### Reconstructed HCE model

Reconstructed HCE tissue (LabCyte CORNEA-MODEL) produced using healthy HCE cells and assay medium were purchased from Japan Tissue Engineering Co., Ltd. (Aichi, Japan). According to the manufacturer’s instructions, this multilayered HCE was fixed in nutrient agar medium for transport.

### Preparation of tissue culture

Reconstructed HCE tissues were placed in 24-well plates filled with 0.5 mL of assay medium warmed to 37 °C, which prevents the formation of air bubbles under the culture inserts. These models were incubated overnight at 37 °C in a 5% CO_2_ incubator, and subsequently transferred into new 24-well plates filled with 0.45 mL of fresh assay medium.

### Details of PM and urban aerosols

PM was collected at Fukuoka University in Fukuoka, Japan, from April 15, 2017 to May 11, 2017 with a high-volume PM sampler using the virtual impactor and cyclone technique without a filter or extraction process^18^. The location of the sampling site is described in detail by Nishita-Hara et al.^52^. This cyclone system had 50% collection efficiency with components that have the following aerodynamic cut-off diameters: virtual impactor, 2.4 µm; FP cyclone, 0.18–0.30 µm; CP cyclone, 0.7 µm. Particles with diameter less than 2.4 µm flowed to the fine side of the virtual impactor. Thus, the particles collected using FP cyclone were approximately 0.30–2.4 µm in aerodynamic diameter (PM_0.3–2.4_), whereas those collected using CP cyclone were larger than approximately 2.4 µm in aerodynamic diameter (PM_>2.4_).

NIES CRM No. 28 UA, which is a certified reference material supplied by the National Institute for Environmental Studies (Ibaraki, Japan), was used as a reference. The origin of UA was atmospheric PM collected on filters placed in a central ventilating system in a building in the centre of Beijing, China. The collection period was 10 years (from 1996 to 2005). The recovered material was sieved using a 32-μm sieve and homogenised. The sieved material was stabilised using cobalt-60 irradiation (2.5 mrad). This UA has a completely different set of characteristics and no equivalency in aerodynamic diameter. However, this UA was used in a similar experiment; some cell lines were exposed to particulate matters^19^, and moreover, another paper recommended using this UA as an analytical quality control and in the evaluation of methods used in the analysis of aerosols, particularly those collected in urban environments in northeast Asia^20^. Therefore, we decided to use UA as a reference.

The particles were suspended in sterile phosphate-buffered saline, ultrasonicated at a concentration of 1 mg/mL, and assessed using a microscope (BZ-9000, Keyence Corporation, Osaka, Japan) at a magnification of 200×. For the cell exposure experiment, the concentration was adjusted to 1, 10, and 100 µg/mL using assay medium, which was supplied by Japan Tissue Engineering Co., Ltd., phosphate-buffered saline, and 0.1% dimethyl sulfoxide.

### Biochemical, chemical, and mineralogical investigations

Endotoxin and β-glucan tests (purchased from Associates of Cape Cod Inc., Falmouth, MA, USA) were performed according to the manufacturer’s instructions. In brief, approximately 1.0 mg of each particle sample was suspended in 0.5 mL water with shaking for 2 min and was then tumbled for 1 h. Each solution was centrifuged briefly, and the supernatants were recovered and endotoxin and β-glucan concentrations determined.

Moreover, the oxidative potential of PMs used in this study was analyzed using dithiothreitol (DTT) assay in accordance with the procedure described by Nishita-Hara et al.^52^. Final DTT activity data were presented as the DTT loss rate normalized by the particle mass in units of picomoles per minute per microgram. These data were calculated by dividing the measured DTT loss rate by the PM mass concentration.

The collected particles were characterized by ion chromatography for anion species (Cl^−^, NO_3_^−^, SO_4_^2−^) and cation species (Na^+^, NH_4_^+^, K^+^, Mg^2+^, Ca^2+^), inductively coupled plasma mass spectrometry (ICP-MS) for elements (Mg, Al, Si, P, S, Cl, K, Ca, Ti, V, Cr, Mn, Fe, Ni, Cu, Zn, Pb), and thermal-optical method (IMPROVE protocol) for OC and EC. The procedure of chemical characterization mentioned above is generally described in several previous papers^53–55^. In addition, the ion data of UA were measured by the above method in this study, and the elements and carbonaceous data are described in several reports^20,53^.

### Experimental protocol

Reconstructed HCE was gently sprinkled with 50 µL of a solution of each particle (PM_0.3–2.4_ and PM_>2.4_ at 1, 10, or 100 µg/mL, and UA at 100 µg/mL) and exposed to these particles for 24 h. We evaluated cell viability, cytokine release and performed histological analysis including immunohistochemical analysis of ZO-1. Three independent experiments were performed to establish UA 100 mg/mL as a reference.

### Determination of cell viability

After 21 h of exposure, 50 µL of Cell Counting Kit-8 (CCK-8) solution (Dojindo Molecular Technologies, Kumamoto, Japan), which utilises water-soluble tetrazolium salt (WST-8), was added to each assay medium, which was stored at 37 °C in a 5% CO_2_ incubator for the remaining 3 h of the 24-h incubation period. WST-8 is reduced by dehydrogenases in cells to obtain an orange-coloured product (formazan), which is soluble in the tissue culture medium. The amount of formazan dye generated in the cells is directly proportional to the number of living cells. Subsequently, assay medium was added to each well of a 96-well plate (200 µL/well), and the optical density of each well was measured at 450 nm using a microplate reader (PowerWave X, BioTek Instruments Inc., VT, USA). The results were expressed as the percentage of the control group (0 µg/mL).

### Cytokine/chemokine analysis

The medium was collected after exposure and stored at − 80 °C. Within 1 week prior to flow cytometry analysis, the concentrations of interleukin (IL)-6 and IL-8 were measured using Multiplex Cytokine Assay kits (Luminex Human Magnetic Assay; R&D Systems, MN, USA) with detection limits of < 1.7 and 1.8 pg/mL, respectively. These Magnetic Luminex Assay multiplex kits are designed as described below. Analyte-specific antibodies are pre-coated onto color-coded magnetic microparticles. These magnetic microparticles that capture the measurement object are fixed as a monolayer on the bottom of the well, and the type of measurement object is identified by irradiating LEDs with different wavelengths, and the signal intensity is measured. These evaluations were performed by Filgen, Inc. (Nagoya, Japan).

### Histological analysis

One reconstructed HCE in each group was rinsed and fixed with 10% formaldehyde solution for histological analysis. The polycarbonate membrane was cautiously cut, and the cultures were embedded in paraffin and sectioned at 4-µm thickness. The sections were deparaffinised and stained with haematoxylin and eosin. These sections were assessed using Moticam Pro 252A (Motic, Hong Kong, China) at a magnification of 100× (divided into three parts: both sides and centre of each model). These evaluations were performed by Kyodo Byori, Inc. (Kobe, Japan). The captured photographs were analysed using WinROOF imaging software (Mitani Shoji Co., Ltd., Fukui, Japan) to determine the area of the tissue (µm^2^).

### Immunohistochemical analysis

For immunohistochemical analysis, paraffin-embedded sections were deparaffinised and heated in 10 mM citrated buffer (pH 6.0) for epitope retrieval. The cultures were incubated with anti-rabbit ZO-1 antibodies (1:100 dilution; Thermo Fisher Scientific, IL, USA) at 4 °C overnight and with fluorescein-labelled anti-rabbit IgG goat polyclonal antibodies (1:500 dilution; Life Technologies, OR, USA) for 1 h at room temperature. The slides were immersed in coverslip mounting medium containing 4 '6-diamidino-2-phenylinodole dihydrochloride (Dojindo, Kumamoto, Japan). The slides were photographed using a BZ-X810 fluorescence microscope (Keyence Corporation, Osaka, Japan) at a magnification of 200× to obtain a complete image of each group. These evaluations were performed by Kyodo Byori, Inc. (Kobe, Japan). The captured photographs were analysed using WinROOF imaging software (Mitani Shoji Co., Ltd., Fukui, Japan) to determine the area of ZO-1 compared with the whole area (%).

### Statistical analysis

All data are presented as mean ± standard error of the mean. One-way analysis of variance, followed by Dunnett’s test (cell viability and IL-8) and Dunnett’s T3 test (Il-6), was used to analyse the significance of differences between the groups using the Statistical Package for the Social Sciences software (IBM Corp., Armonk, NY, the USA). A *p* value of < 0.05 was considered statistically significant.

## Data Availability

All data generated or analysed during this study are included in the published article.
